# Trimester-specific reference interval for glycated hemoglobin in non-diabetic pregnant women: A cross-sectional study

**DOI:** 10.1016/j.clinsp.2026.101160

**Published:** 2026-09-19

**Authors:** Kassahun Takiso, Aster Tsegaye, Mekoya Dabulo, Abenet Woldesenbet, Abebaye Leminie

**Affiliations:** a Department of Medical Physiology, Addis Ababa University, Addis Ababa, Ethiopia; b Department of Medical Laboratory Science, Addis Ababa University, Addis Ababa Ethiopia; c Department of Obstetrics and Gynecology, Addis Ababa University, Addis Ababa, Ethiopia

**Keywords:** Ethiopian, Non-diabetic pregnant women, Glycated hemoglobin, Glycemic control, Pregnancy trimester, Reference interval

## Abstract

**Background:**

Gestational Diabetes Mellitus (GDM) is a growing public health concern in Ethiopia. HbA1c is useful for assessing glycemia but is influenced by physiological changes in pregnancy. To our knowledge, no published trimester-specific HbA1c reference intervals exist for Ethiopian non-diabetic pregnant women. This limits population-specific clinical interpretation.

**Methods:**

A facility-based cross-sectional study was conducted from February to July 2025 among 424 non-diabetic pregnant women across all trimesters. Normality of HbA1c distribution was assessed using the Shapiro-Wilk and Kolmogorov-Smirnov tests; outliers were identified using Tukey’s fences method. Reference intervals were determined parametrically (mean ±2SD). Differences across trimesters were tested using one-way ANOVA. Because Levene’s test indicated a violation of the homogeneity of variances (p < 0.001) and group sizes were unequal, pairwise comparisons were performed using the Games-Howell post hoc test. A p-value < 0.05 was considered statistically significant.

**Results:**

HbA1c showed a normal distribution across all trimesters. Reference intervals were 4.8–5.9% in the first, 4.2–5.8% in the second, and 4.5–5.9% in the third trimester. Mean HbA1c levels differed significantly between trimesters (p < 0.05). Lowest in the second trimester 5.00% (95% CI 4.93–5.07) and highest in the first trimester 5.35% (95% CI: 5.31–5.39). Games-Howell post hoc tests confirmed that all pairwise comparisons were significant (p < 0.05).

**Conclusion:**

Trimester–specific HbA1c reference intervals were defined for non-diabetic pregnant women, demonstrating measurable physiological variation across gestation. The use of locally derived thresholds could improve the accuracy of GDM screening and support more appropriate clinical interpretation.

## Highlights

• First trimester-specific glycated hemoglobin intervals in Ethiopia.
• Glycated hemoglobin lowest in mid-pregnancy, rises in late pregnancy.
• Study of 424 healthy pregnant women across all trimesters.
• Supports glycemic assessment where glucose testing is limited.

## Introduction

Diabetes Mellitus (DM) is a metabolic disorder which causes hyperglycemia due to defects in insulin secretion, insulin action, or both. There are currently five types diabetes includes: Type 1 diabetes, Type 2 diabetes, gestational diabetes, a hybrid form of diabetes, and other specific types of diabetes.^1^ Gestational Diabetes Mellitus (GDM) is generally considered to be hyperglycemia or impaired glucose tolerances that occur during pregnancy.^2^

GDM is responsible for up to 90% of diabetes complications in pregnancy and is a risks factor for macrosomia, preeclampsia, cesarean delivery, and neonatal complications.^3^ The prevalence of GDM is estimated to be around 14% in the world. A recent systematic review and meta-analysis of 59 studies from 16 countries in African reported that the pooled prevalence of GDM was 14.0% (95% CI 11.6–16.5%) with the lowest prevalence in Southern Africa at 10.2% and highest prevalence in Central Africa at 18.0%. The overall incidence of GDM in Eastern Africa was 13.9%, highlighting the high burden of GDM in the region.^4^ The pooled prevalence in Ethiopia around 12.04%^5^ and a higher prevalence of 16.9% were reported among facility in Addis Ababa.^6^

Many laboratory tests exist for the diagnosis and treatment of DM, however, their consistency and usefulness is not conclusive. Optimal glycemic control remains the cornerstone of GDM management; however, there is no global consensus on the most appropriate diagnostic criteria or the most effective screening approach.^7^ The standard Oral Glucose Tolerance Test (OGTT) used for diagnosing GDM is labor-intensive, costly, and inconvenient for pregnant women. In addition, studies have reported poor acceptability and tolerability of the OGTT, which may contribute to incomplete testing and high dropout rates, particularly in resource-limited settings.^8^

In this context, glycated Hemoglobin (HbA1c) has been proposed as a complementary marker because of several methodological advantages, including that it does not require fasting, exhibits low biological variability, and is generally more convenient and acceptable to pregnant women. However, a recent meta-analysis concluded that HbA1c alone is insufficient for GDM screening and should be used in combination with other clinical risk factors or biomarkers to improve screening performance.^9^ Biochemically, HbA1c is a minor variant of Hemoglobin A (HbA) formed through the non-enzymatic attachment of glucose to the N-terminal valine of the beta-globin chain. It reflects the average blood glucose level over the preceding 2 to 3 months, making it a valuable indicator of long-term glycemic control.^10^

In Ethiopia, the development of locally derived reference intervals for HbA1c has been limited. Consequently, HbA1c results are frequently interpreted using manufacturer-provided reference intervals derived from non-Ethiopian populations, which may not accurately reflect the local population. A recent Ethiopian study establishing reference interval for non-diabetic women highlighted this important gap and emphasized the need for population specific reference interval to improve clinical interpretation.^11^

However, reference intervals established for non-diabetic pregnant adults cannot be directly applied to pregnant women because pregnancy is accompanied by physiological and hematological adaptations that influence HbA1c concentrations independent of glycemia. These changes vary across gestation, emphasizing the need for trimester-specific HbA1c reference intervals.^12^^,^^13^ Therefore, this study aimed to establish trimester-specific reference intervals for HbA1c among non-diabetic pregnant Ethiopian women.

### Statement of significance

Despite the wide spread use of HbA1c for assessing long-term glycemic control, the application of a single cutoff value during pregnancy may lead to misclassification of glycemic status due to physiological and hematological changes that occur across gestation. In addition, HbA1c levels are influenced by population-specific factors, including race and ethnicity, limiting the generalizability of existing reference interval.

Therefore, this study was conducted to establish trimester-specific HbA1c reference intervals among non-diabetic pregnant women in Ethiopia and to determine whether HbA1c reference intervals differ significantly across the first, second, and third trimesters, reflecting physiological changes during pregnancy.

## Materials and methods

### Study design

Cross-sectional study was used to determine trimester-specific reference intervals for non-diabetic pregnant women using HbA1c. The Strengthening the Reporting of Observational Studies in Epidemiology (STROBE) guidelines were used for the design and reported of the study.

### Study setting and period

The study was conducted in Janmeda and Teklehaymanot Health Centers in Addis Ababa, Ethiopia, from February 4 to July 18, 2025. The centers are representative of a diverse ethnic population.

### Study population

Pregnant women who attended their Antenatal Care (ANC) at the health centers in the study period and fulfilled the inclusion criteria were consecutively selected. Non-diabetic pregnant women aged 18 and over attending their Antenatal Care (ANC) in the target health centers during the study period were sequentially recruited if they met the eligibility criteria. The trimester classification was based on the national Ministry of Health guidelines and the clinical definition in the health facilities: first trimester (≤ 12 weeks + 6-days); second (13-weeks 0-days – 27-weeks + 6-days); and third (≥ 28 weeks 0-days – term).

### Eligibility criteria

#### Inclusion

Non-diabetic pregnant women ≥ 18-years of age with a confirmed singleton pregnancy who had attended ANC at one of either of the study sites during the study period.

#### Exclusion

Women who have known Type 1 or Type 2 DM, or who meeting the American Diabetes Association (ADA) criteria for overt diabetes in the first trimester (fasting glucose ≥ 126 mg/dL or HbA1c ≥6.5% [48 mmoL/moL]).^14^ More exclusion criteria were anemia, history of GDM, multiple pregnancy, miscarriage, voluntary termination, and incomplete assessment of GDM. Anemia was defined based on the 2024 World Health Organization (WHO) guideline which recommends hemoglobin thresholds of < 11.0 g/dL and < 10.5 g/dL for the 1st and 2nd trimester, respectively, and of < 11 g/dL in the third trimester.^15^ Other exclusion criteria for women included chronic diseases of hematologic, hepatic, renal, cardiovascular, or immune function.^16^ Also excluded were women who had an alcohol use disorder or a known psychiatric illness.

### Variables and measurements

• Outcome Variables: Glycated hemoglobin (HbA1c, %) measured from venous blood.
• Exposure Variable: Pregnancy trimester (first: ≤ 12-weeks + 6-days, second: 13-weeks 0-days– 27-weeks + 6-days, and third: ≥ 28-weeks 0-days until term).
• Predictor Variables:
  ○ Maternal parameters: Age (years), Weight (Kg), Body Mass Index (BMI, kg/m^2^), systolic and diastolic blood pressure (mmHg).
  ○ Obstetric History: Gravidity and parity (nulliparous, primiparous, multiparous, or grand multiparous).
  ○ Hematological and glycemic parameters: Hemoglobin (g/dL), Fasting Blood Sugar (FBS) (mg/dL), and 2-hour post-load glucose (mg/dl) and 1-hour post-load glucose (mg/dl) of the 75 *g* OGTT for the 2nd and 3rd trimester.
• Diagnostic Criteria:
  ○ GDM diagnosis was done according the criteria of the International Association of Diabetes and Pregnancy Study Groups (IADPSG).^17^ A 75 *g* OGTT was used to diagnose GDM if the plasma glucose level at any point during the test met or exceeded one of the following thresholds: fasting glucose level of 92 mg/dL or more (5.1 mmoL/L or more), 1-hour glucose level of 180 mg/dL or more (10.0 mmoL/L or more), or 2-hour glucose 153 mg/dL or more (8.5 mmoL/L or more).

### Data collection and laboratory procedures

Standardized instruments and procedures were used to collect data by trained health professionals.

### Demographic and clinical data

Maternal age, weight, height, obstetric history, medical and family history of diabetes was collected using a semi-structured, interviewer-administered questionnaire. BMI was defined as weight (kg)/height (m^2^). The last menstrual period was used to determine gestational age, which was confirmed by ultrasound.

### Blood pressure

Measured using a calibrated digital sphygmomanometer after a 5-minute rest in a seated position.

### Hemoglobin (Hb)

Venous whole blood (3 mL) was collected in EDTA tubes and analyzed immediately using the Sysmex XS-500i automated hematology analyzer (sodium lauryl sulfate method).

### HbA1c

EDTA blood samples were transported to Black Lion Hospital Laboratory and analyzed within 2‒3 h using the Roche Cobas C 311 analyzer, based on the Turbidimetric Inhibition Immunoassay (TINIA).

According to the manufacturer’s documentation and FDA 510(k) summary, the assay demonstrates no clinically significant interference (bias within ±7%) from common hemoglobin variants, including HbS, HbC, HbD, and HbE, within clinically relevant concentrations. However, elevated fetal hemoglobin (HbF > 7%) has been reported to cause significant negative interference.^18^

### FBS and OGTT

Venous blood (2 mL) was collected after a fasting period of 8–12 h; Participants were instructed to fast for at least 8–12 h prior to sample collection in accordance with standard clinical procedures. Fasting status was confirmed verbally at the time of collection. For the second and third trimesters, this served as a baseline for the 75 g OGTT. Subsequent samples were collected at 1-hour and 2-hour intervals.

### Quality assurance

The same instruments, reagents, and laboratory technologists were used across all three trimesters and study sites to ensure comparability.

### Bias control

Consecutive enrollment of all eligible women that attending Antenatal Care (ANC) in the study period reduced selection bias. Standardized, pretested protocol and automated analysis with tight internal quality control procedures reduced measurement bias. Potential confounding factors were controlled for by applying pre-established exclusion criteria as above.

The results revealed that with regard to iron and folic acid supplement, supplementation was initiated in the 1st trimester and not routinely continued in other trimesters as per national ANC guideline. Blood samples were taken to reduce the potential confounding effect of the supplement, before supplement (median gestational age: 8-weeks).

### Sampling technique

The screening for eligibility of all non-diabetic pregnant women who attend ANC in any trimester at Janmeda and Teklehaymanot Health Centers was undertaken between February 4th, 2025 and July 18th, 2025. Eligibility participants were consecutively recruited for each trimester (first, second, or third) until the minimum recruited sample size for each trimester was achieved or slightly exceeded.

### Sample size

Sample size was calculated based on the recommendations of the International Federation of Clinical Chemistry and Laboratory Medicine (IFCC) recommendations. The sample size for comparisons between several groups is recommended to be 40 participants per group^19^ and direct non-parametric reference intervals estimation is recommended to be at least 120 reference individuals per group.^20^ No information on the distribution of the HbA1c results was available prior to the study, so a minimum sample size of 120 per trimester was established. After data collection and prespecified outlier exclusion, the HbA1c data were found to be approximated normally distributed so, the parametric approach was used to estimate the reference intervals.

### Statistical analysis

The IBM SPSS Statistics Version 27 software was used for data analysis. Normality of HbA1c distribution in the trimesters was evaluated with the Shapiro-Wilk and Kolmogorov-Smirnov tests.

Normality was determined both before and after the removal of outlier. Tukey’s Fences method^21^ was used to detect outliers. 4, 3, and 2 outliers were removed from the first, second, and third trimesters, respectively. The distribution of HbA1c following exclusion resembled normal distribution. HbA1c levels were approximated to be normally distributed after predefined exclusion of outliers. Hence, parametric methods (mean ±2 SD) were used to estimate reference interval. This method follows the recommendations of CLSI EP28-A3c, which recommends parametric estimation if after appropriate data assessment, the reference data are approximately normally distributed. Non-parametric reference intervals were also determined as sensitivity analysis to assess the robustness of the primary parametric estimates. The median and Interquartile Ranges (IQR) were used to summarize continuous variables that are not normally distributed. Levene’s test was performed to check homogeneity of variances. One-way ANOVA with Games-Howell post hoc tests (which do not assume equal variances) were used because the data obtained did not meet the criteria of homogeneity and equal group sizes. A p-value < 0.05 was considered statistically significant. Those who had missing, or other important variables were not included in the analysis.

## Results

### Participants

524 non-diabetic pregnant women were screened for eligibility. Following the exclusion of those who met the exclusion criteria, 100 women were excluded due to any of the following reasons: known type 1 or 2 DM (n = 4), overt diabetes (n = 5), previous history of GDM (n = 3), newly diagnosed GDM (n = 42), anemia (n = 2), failure to complete OGTT (n = 41), and other medical conditions (n = 3).

The prevalence of GDM was found to be 16.15% among those who were screened in the 2nd and 3rd trimester. Therefore, 424 non-diabetic pregnant women final analyzed to determine trimester-specific reference intervals for HbA1c. Enrollment of the participants is presented in Fig. 1.

**Fig. 1:**
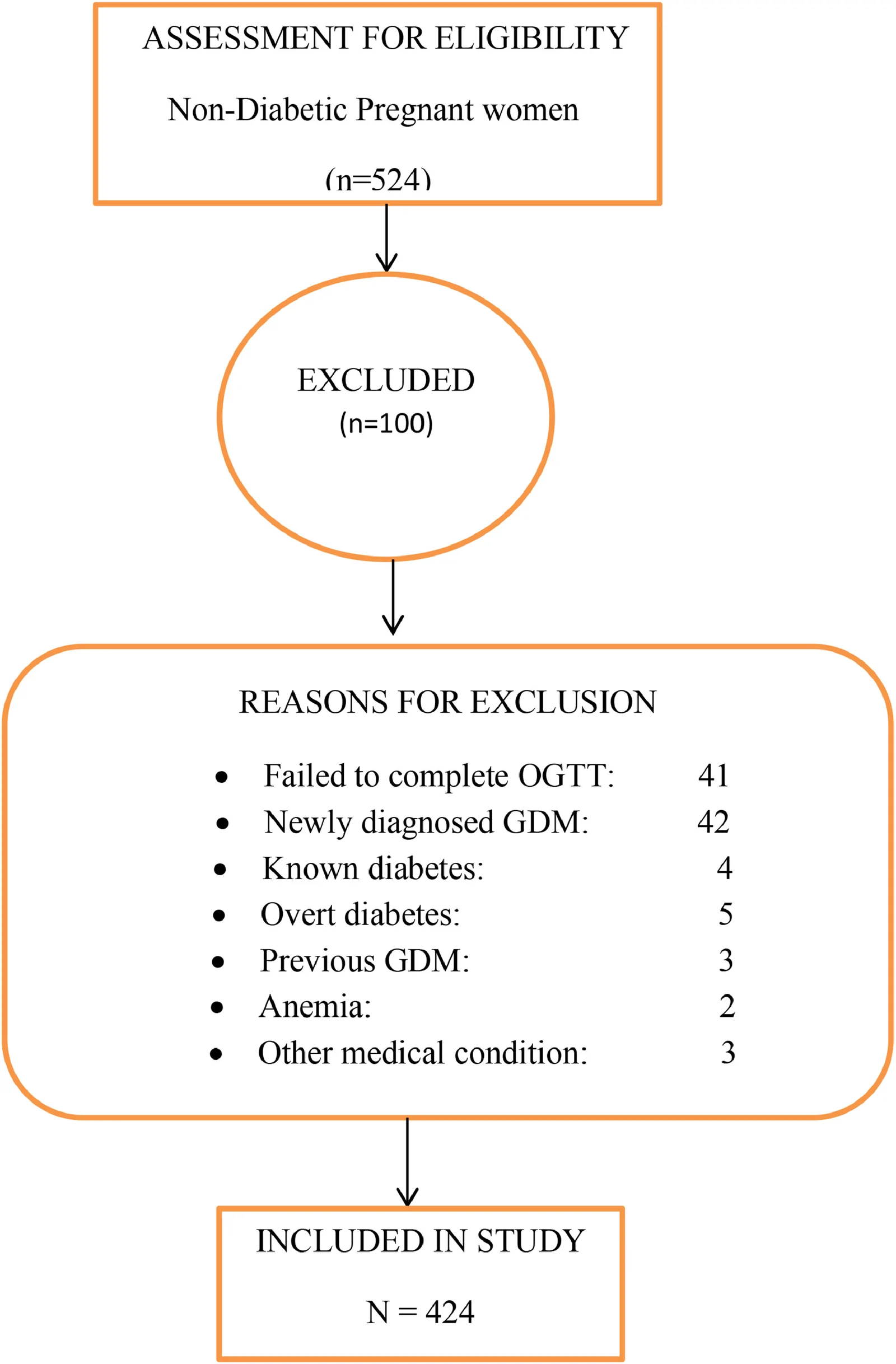
Study participant enrollment flowchart.

### Maternal clinical characteristics and glucose measurements

424 women were enrolled, 164 in the first, 139 in the second, and 121 in the third trimester. No data were missing.

The value of maternal weight, BMI, and gestational age in each trimester group were clinical expected range. Systolic and diastolic blood pressures were lowest at the 2nd trimester and a slightly higher at the 3rd trimester. Later trimester groups had lower hemoglobin concentrations than earlier trimester groups. The third-trimester group had lower fasting blood glucose level than the second trimester group. While the OGTT 1-hour value was slightly higher in the third-trimester group compared with the second-trimester group, and the 2-hour value was similar between the second-and third-trimester group.

The values of the OGTT were not normally distributed and therefore compared in the second and third trimesters using Mann-Whitney *U test*, as the data. The 1-hour OGTT was significantly elevated in the third trimester as compared with the second trimester (161 mg/dL, IQR 154 to 168 vs. 156 mg/dL, IQR 149 to 163, respectively: Mann-Whitney *U* = 5636.5, p < 0.001). In contrast, the 2-hour OGTT showed no significant difference between the second (median 135 mg/dL, IQR 129.5–140.5) and third trimesters (median 136 mg/dL, IQR 132–140) (Mann-Whitney *U* = 7935.5, p = 0.432) (Table 1).

**Table 1:** Maternal clinical and anthropometric characteristics by trimester (non-diabetic pregnant women, ethiopia).

Variable	First Trimester (n = 164)	Second Trimester (n = 139)	Third Trimester (n = 121)
Gestational age (Weeks)	8.0 (7.7–10.4)	26 (25–27)	30 (29.4–30.7)
Age (years)	27 (26–30)	29 (26–36)	28 (26–30)
Weight (kg)	55 (50–62)	61 (56–67)	63 (56.5–68.5)
BMI (kg/m^2^)	22.9 (21.3–25.3)	25.1 (23.1–27.1)	25.5 (23.4–27.7)
Systolic BP (mmHg)	108 (100–113)	99 (92–108)	106 (98.5–113.5)
Diastolic BP (mmHg)	67 (62–72)	61.9 ± 6.4	68 (63–73)
Hemoglobin (g/dL)	13.51 ± 0.97	12.8 (12.2–13.4)	12.6 (12.1–13.2)
FBS (mg/dL)	96 (88–100)	85 (80.5–89.5)	82 (76–87)
1-hour OGTT (mg/dL)	NA	156 (149–163)	161 (154–168)
2-hour OGTT (mg/dL)	NA	135 (129.5–140.5)	136 (132–140)

OGTT was not performed in the first trimester, Data distribution was assessed using normality tests: normally distributed variables presented as mean (SD), while skewed variables are presented as median (Interquartile Range [IQR]: 25th‒75th percentile).

BMI, Body Mass Index; BP, Blood Pressure; FBS, Fasting Blood Sugar; OGTT, Oral Glucose Tolerance Test; NA, Not Applicable.

### Trimester-specific HbA1c reference intervals

After exclusion of statistical outliers identified using Tukey’s fences, HbA1c values were normally distributed across all trimesters, as confirmed by Kolmogorov-Smirnov and Shapiro-Wilk tests (all p > 0.05; see Table 2).

**Table 2:** Tests of normality for trimester-specific HbA1c values.

Trimesters	n	Kolmogorov-Smirnov	p-value	Shapiro-Wilk	p-value
		Statistic		Statistic	
First	164	0.039	>0.200	0.993	0.558
Second	139	0.066	>0.200	0.988	0.271
Third	121	0.051	>0.200	0.981	0.078

A p-value > 0.05 indicates no significant deviation from normality.

In contrast, prior to outlier exclusion, HbA1c distribution deviated from normality in all trimesters (Shapiro-Wilk test, p < 0.05).

Based on this normal distribution of HbA1c values, parametric methods were applied to estimate trimester-specific reference intervals. The resulting intervals (mean ± 2SD) were 4.8–5.9% (First trimester), 4.2–5.8% (Second trimester), and 4.5–5.9% (Third trimester) (Table 3).

**Table 3:** Trimester-specific reference interval for HbA1c (non-diabetic pregnant women).

Trimester	n	Mean (%)	SD	95% CI of mean (%)	Reference Interval (%)
First trimester	164	5.35	0.269	5.307–5.390	4.8–5.9
Second trimester	139	5.00	0.404	4.932–5.068	4.2 –5.8
Third trimester	121	5.20	0.352	5.137–5.264	4.5–5.9

Reference intervals were calculated parametrically (Mean ± 2SD).

However, in accordance with CLSI EP28-A3c recommendations, non-parametric methods (2.5th–97.5th percentile) were also calculated as a sensitivity analysis to compare with the primary parametric approach. Given the adequate sample size in the first trimester (n = 164 ≥ 120), non-parametric intervals are reported for comparison only.

For the first-trimester, the non-parametric reference interval (2.5th–97.5th percentile) was 4.9–5.8%, while the parametric interval was 4.8–5.9%. The difference between the upper limits of the two methods was 0.1%, which is clinically negligible (< 0.2% HbA1c). A comparison of both methods is presented in (Table 4).

**Table 4:** Comparison of parametric and non-parametric reference intervals for HbA1c in the first trimester (n = 164).

Method	Lower Limit (%)	Upper Limit (%)
Non-parametric (2.5th –97.5th	4.9	5.8
Parametric (Mean ± 2SD)	4.8	5.9

The small difference (0.1%) is clinically negligible, and the primary parametric intervals are reported throughout the manuscript.

For the third trimester, the non-parametric reference interval (2.5th–97.5th percentile) was 4.5–5.8%, compared with the primary parametric interval (mean ± 2SD) of 4.5–5.9%. The difference between the upper limits was 0.1% (parametric 5.9% vs. non-parametric 5.8%), which remains within the clinically negligible threshold (≤ 0.2% HbA1c) (Table 5).

**Table 5:** Comparison of parametric and non-parametric reference intervals for HbA1c in the third trimester (n = 121).

Method	Lower Limit (%)	Upper Limit (%)
Non-parametric (2.5th–97.5th	4.5	5.8
Parametric (Mean ± 2SD)	4.5	5.9

After outlier exclusion based on the analytical criteria, HbA1c levels did not show significant deviation from the normal limits in all trimesters (Shapiro-Wilk p > 0.05). Thus, the mean ± 2SD (parametric) was kept as the default analytic method, following the CLSI EP28-A3c guidelines for approximate Gaussian distribution. Additionally, non-parametric intervals were computed as a sensitivity analysis to assess the robustness of the estimated intervals. The observed difference between methods was minimal (≤ 0.1% HbA1c), not clinically relevant, indicating that the primary parametric intervals were stable and not materially influenced by analytical method selection.

Sensitivity analysis (with and without outliers’ exclusion) revealed modest differences, primarily affecting the upper reference limits. Inclusion of outliers slightly widened the intervals but did not alter the overall clinical interpretation of trimester-specific HbA1c patterns.

### Comparison of HbA1c across trimesters

Levene’s test indicated a violation of homogeneity of variances (*F* = 10.827, p < 0.001). Given unequal group sizes, Games-Howell Post hoc tests were used for pairwise comparisons.

Mean HbA1c levels differed significantly across trimesters (one-way ANOVA, *F*(2, 421) = 39.227, p < 0.001). The second trimester had the lowest mean (5.00%), followed by the third trimester (5.2%) and first trimester (5.35%) (Table 6). Games-Howell Post hoc tests confirmed significant differences between all pairs (p < 0.05), indicating the order: second < third < first trimester.

**Table 6:** Mean HbA1c levels across trimesters (ANOVA and post hoc analysis, non-diabetic pregnant women, ethiopia).

Group	n	Mean HbA1c (%)	95% CI of Mean	ANOVA/post hoc results
First trimester	164	5.35	5.31–5.39	a
Second trimester	139	5.00	4.93–5.07	b (lowest)
Third trimester	121	5.20	5.14–5.26	c
**Test**			**Result**	
Overall ANOVA			F (2, 421) = 39.227, p < 0.001	
Levene’s test (homogeneity)			*F* = 10.827, p < 0.001	
Post-hoc test			Games-Howell	

Groups with different letters (a, b, c) are significantly different at p < 0.001 (Games- Howell post hoc. Order: second < third < first trimester.

### Precision of trimester-specific HbA1c reference interval

The precision of the trimester-specific HbA1c reference intervals was evaluated by calculating Confidence Intervals (CIs) for the reference limits in accordance with CLSI EP28-A3c guidelines. For the first, second, and third trimesters, the 95% CIs around both lower and upper reference limits were narrow. Specifically, the 95% CIs ranging from 4.74 to 4.86 and 5.84–5.96 in the first trimester, 4.10–4.30 and 5.70–5.90 in the second trimester, and 4.42% to 4.58% and 5.82–5.98 in the third trimester from the lower and upper limits, respectively. These findings indicate good statistical precision of the estimated reference intervals across all trimesters (Table 7).

**Table 7:** Precision of trimester-specific HbA1c reference intervals: 95% confidence intervals for reference limit.

Trimester	n	Lower Reference Limit (%)	95% CI of Lower Limit (%)	Upper Reference Limit (%)	95% CI of Upper Limit (%)
First	164	4.80	4.74–4.86	5.90	5.84–5.96
Second	139	4.20	4.10–4.30	5.80	5.70–5.90
Third	121	4.50	4.42–4.58	5.90	5.82–5.98

Note: Reference limits were estimated using the parametric methods (Mean ± 2SD). Ninety-five percent confidence intervals for the reference limits were derived using parametric methods. (n) Represents the number of participants.

### Pairwise comparisons of HbA1c between trimesters (games-howell post-hoc, non- diabetic pregnant women)

Games-Howell post hoc tests (which do not assume equal variances) confirmed significant pairwise differences in HbA1c between trimesters. The largest difference was observed between the first and second trimesters (mean difference 0.3487%, 95% CI: 0.254 to 0.443, p < 0.001), followed by first vs. third trimester (mean difference 0.1479%, 95% CI: 0.057 to 0.238, p < 0.001) and second vs. third trimester (mean difference −0.2008%, 95% CI: −0.311 to −0.090, p < 0.001), demonstrating a non-linear trend with lowest HbA1c in the second trimester (Table 8).

**Table 8:** Pairwise comparisons of HbA1c between trimesters (games-howell post-hoc, non- diabetic pregnant women).

Comparison	Mean Difference (%)	95% CI	p-value
First vs. Second	0.3487	0.254 to 0.443	<0.001
First vs. Third	0.1479	0.057 to 0.238	<0.001
Second vs. Third	−0.2008	−0.311 to −0.090	<0.001

Games-howell post-hoc test was used due to violation of homogeneity of variance (Levene’s *F* = 10.827, p < 0.001).

### Comparison of HbA1c distribution between trimester groups

Box-and-whisker plots illustrate the distribution of HbA1c values across pregnancy trimesters. Mean HbA1c differed significantly among the trimester groups (One-way ANOVA, p < 0.001), with the highest values observed in the first trimester, the lowest in the second trimester, and intermediate values in the third trimester (Fig. 2).

**Fig. 2:**
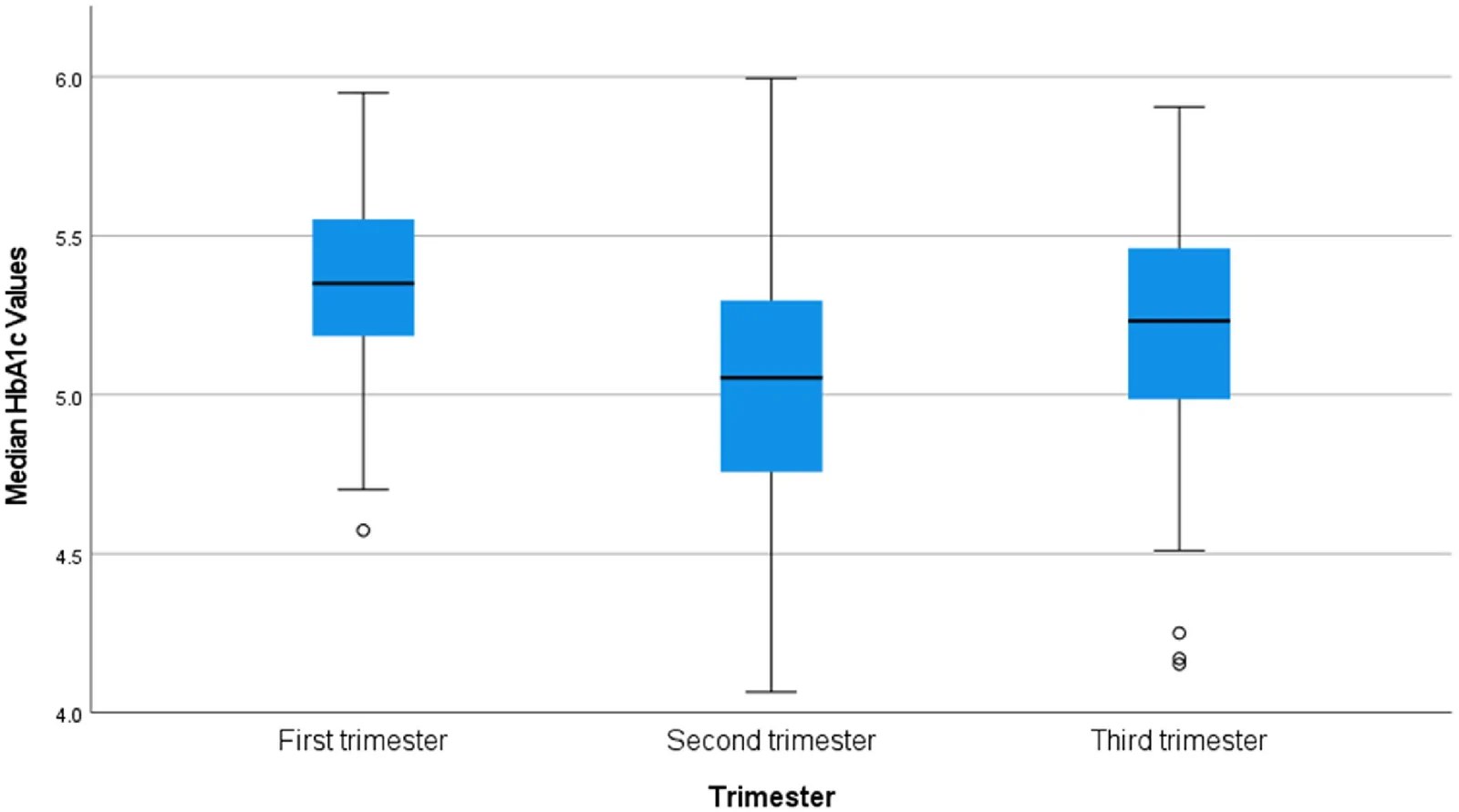
Box-and-whisker plots showing the distribution of HbA1c values across pregnancy trimesters. The boxes represent the median and interquartile range, and the whiskers represent the range of observations. Statistical comparisons of HbA1c between trimester groups were performed using one-way ANOVA, which demonstrated significant differences among the group means (p < 0.001). Median HbA1c was highest in the first trimester, lowest in the second and intermediate in the third, indicating statistically significant differences between trimester groups (p < 0.001, one-way ANOVA).

## Discussion

This study offers the trimester-specific reference intervals of HbA1c for non-diabetic pregnant women in Ethiopia. Results showed that there were significant differences in HbA1c values between the trimesters: lower mean values were seen in the second trimester (mean 5.00%, RI: 4.2–5.8%) than in the first (mean 5.35%, RI: 4.8–5.9%) and greater mean value was seen in the third trimester (5.20%, RI: 4.5–5.9%) than in the second trimester. The difference in HbA1c levels between groups of non-diabetic women at different gestation periods were all statistically significant (p ≤ 0.001).

The HbA1c levels were lower in the second trimester than the first, reflecting the period of increased glucose demand by the fetoplacental unit, which reduces maternal glucose concentrations. In addition, during pregnancy, an increased proportion of erythrocytes with a decreased lifespan and shorter time for glycation also contribute to decreased HbA1c levels. 22, 23, 24, 25 This physiological decline in hemoglobin concentration across gestation has also been reported in recent African populations. For example, Simpong et al. (2023) observed a progressive decrease in median hemoglobin from 11.8 g/dL in the first trimester to 11.0 g/dL and 10.5 g/dL in the second and third trimesters, respectively. ^26^

The higher HbA1c in the third trimester than the second trimester was in contrast to the rise in insulin resistance that happens during late pregnancy. In this time period, placental and maternal hormones such as estrogen, progesterone, cortisol, leptin, placental lactogen and placental growth hormone gradually decrease insulin sensitivity, which results in an increased level of glucose and related HbA1c levels. ^27^^,^^28^ Recent studies also have reported that placental IGFBP1 is a potentially important regulator of insulin sensitivity during pregnancy. ^29^

Median FBS values decreased throughout gestation, from 96 mg/dL in the first trimester to 85 mg/dL and 82 mg/dL in the second and third trimesters, respectively. The magnitude of decline (14 mg/dL from first to third trimester) however seemed greater than has previously reported. ^30^^,^^31^

In order to assess if this was due to this methodology or a true physiological effect, the second/third-trimester exclusion criterion for GDM (FBS ≥ 92 mg/dL) was applied to the first-trimester cohort and a sensitivity analysis was performed. The 164 first-trimester participants included 95 (57.9%) with FBS ≥ 92 mg/dL who were excluded. The median FBS in the first-trimester dropped from 96 mg/dL to 86 mg/dL after the exclusion, and the difference between the first and third-trimester dropped from 14 mg/dL to 4 mg/dL. This confirms that differential exclusion criteria across trimesters ‒ rather than true physiological change ‒ were the primary driver of the apparent large decline. After harmonizing exclusion criteria, the residual decline of 4 mg/dL aligns with physiologically expected values reported in the literature, ^30^^,^^31^ These findings underscore the importance of consistent exclusion criteria when comparing metabolic parameters across trimesters in cross-sectional studies. ^32^

It is important to emphasize a key interpretative consideration of this study. Because the analysis is cross-sectional, the observed differences between trimester groups reflect comparisons between different individuals rather than changes within the same individuals over time. Therefore, these findings cannot be interpreted as evidence of a temporal pattern, trajectory, or physiological progression of HbA1c during pregnancy. These differences observed may be real physiological variations, but may also be due cohort effects or selection bias or residual confounding from unobserved differences between groups. Establish within-individual changes on HbA1c can only be done with longitudinal studies following the same women during pregnancy.

Our findings are comparable to results reported in longitudinal and international studies, but should be interpreted as between-group differences rather than evidence of within-individual physiological change.

The lower level of HbA1c in the second than in the first and third- trimester groups is similar to that observed in China, ^33^ India, ^34^ and the Netherlands. ^35^

Importantly, direct quantitative comparison of absolute HbA1c values across these international studies is methodologically constrained. As detailed in our Material and Methods section, our study employed the Roche Cobas C 311 analyzer using a Turbidimetric Inhibition Immunoassay (TINIA), which, according to the manufacturer’s documentation, demonstrates significant negative interference when fetal Hemoglobin (HbF) exceeds 7% (Table 2). The comparator studies used different analytical platforms (e.g., HPLC in the Chinese study, ^33^ immunoassay in the Japanese study ^36^ and inter-assay variability is well-documented in the literature. ^37^ Furthermore, differences in exclusion criteria (e.g., our exclusion of women with hemoglobin < 11 g/dL vs. variable definitions of anemia in other studies), trimester definitions (ours first trimester: ≤ 12-weeks +6-days, second trimester: 13-weeks 0-days – 27-weeks + 6-days, third trimester: ≥ 28-weeks 0 days until term vs. variable cutoffs in other studies), and statistical approaches (our parametric method after outlier removal vs. non-parametric methods in some comparator studies) preclude valid statistical comparison or equivalence testing. Without harmonization to a common standard (e.g., IFCC reference method), such comparisons remain descriptive at best. ^38^

With these methodological limitations in mind, we note that the absolute HbA1c values of our cohort are fairly consistent with the Japanese ^36^ and Mexican ^39^ cohorts. However, this observation is descriptive and hypothesis-generating rather than confirmatory. This pattern likely reflects population-specific influences, including differences in dietary intake, ethnicity and genetic background, all of which may affect HbA1c independently of glycemia. These observations support the use of locally derived reference intervals rather than the direct adoption of values from other populations ‒ a conclusion reinforced by the demonstrable methodological heterogeneity across studies.

Also, hemoglobin variants may need to be taken into account when interpreting HbA1c levels. An epidemiological estimate from global sources indicates that the prevalence rate of abnormal hemoglobins, such as thalassemia and sickle cell trait are relatively low in Ethiopia (< 4%) in Ethiopia. ^40^ However, there is a lack of contemporary, population-specific data, and importantly, no studies have reported the prevalence of these hemoglobin variants specifically among Ethiopian non-diabetic pregnant women. Given that such variants may affect HbA1c measurement and interpretation, their unmeasured presence represents a potential limitation. Nevertheless, considering the relatively low estimated prevalence, their overall impact on the present findings is likely minimal.

Environmental factor may also play a role. Addis Ababa is located at high altitude (∼2355 m above the sea level), ^41^ where chronic hypobaric hypoxia induces adaptive change in erythropoiesis, erythrocytes turnover, and hemoglobin concentration. ^42^ Such hematologic adaptations can influence HbA1c formation and its relationship with average glucose. Previous studies in high altitude-setting have reported differences in HbA1c-fasting glucose concentrations and, in some case, higher optimal HbA1c thresholds compared with sea level population. ^43^

Several studies have directly examined the relationship between HbA1c and altitude. Bazo-Alvarez et al. (2017) reported that HbA1c levels in non-diabetic adults decreased with increasing altitude in Peru, independent of glucose levels, suggesting that attitude-related erythrocytosis affects HbA1c measurements. ^44^ Similarly, Ren et al. (2020), in a population based study conducted in an oxygen-deficient plateau, demonstrated that high-altitude erythrocytosis influences the performance of HbA1c for detecting diabetes, independent of glucose levels. ^45^ Zhou et al. (2017) compared HbA1c concentrations between sea-level and high-altitude populations in China and found differences that could not be explained by glucose alone. ^46^ Most relevant to our study population, Cui JH et al. (2022) examined HbA1c in late pregnancy on the Tibetan Plateau and reported that altitude adjustment significantly affect anemia prevalence estimates, further supporting the influence of altitude on HbA1c interpretation in non-diabetic pregnant women. ^47^ Although women with anemia were excluded from our study, we can note exclude the possibility that chronic hypoxic exposure and associated hematological adaptations at high altitude may influence HbA1c levels. However, we acknowledge that hematologic parameters (e.g., red blood cells count, mean corpuscular volume, and reticulocyte count) were not measured; therefore, altitude-related hematologic effects could not be directly assessed in this cohort. Consequently, this remains a hypothesis that should be evaluated in future studies incorporating comprehensive hematologic profile.

Nonetheless, our findings of trimester-specific HbA1c reference intervals in this high-altitude population provide a basis for future comparative studies integrating both hematologic and glycemic parameters. These altitude-related factors may partly explain the observed differences in HbA1c intervals compared with low-altitude populations, such as those reported in the Netherlands and Japan. Considering of altitude therefore adds important context to the interpretation of population-specific HbA1c reference intervals.

A key observation in our study is the statistical significance of HbA1c differences across all trimesters – a consistency not universally observed elsewhere. For instance, both our study and ^33^ reported significant pairwise differences between all trimesters (p < 0.001). In contrast, ^36^ in Japan and ^39^ in Mexico City found no significant differences between the first and third trimesters (p > 0.05).

A comparison of Caucasian and Asian cohorts showed that difference between the second and third trimesters groups reached statistical significance only among Caucasian women, suggesting that although the direction of HbA1c differences is broadly consistent, the magnitude and statistical detectability may vary across populations.

Evidence from South Asian is consistent with these findings. A study from India ^34^ demonstrated lower absolute HbA1c levels but similar differences between trimester groups, with significant differences between the first and second trimester (p < 0.05). In contrast, a study from Pakistan ^48^ reported lower absolute reference intervals and did not assess statistical differences between groups.

Methodologically, our use of a parametric approach is comparable with studies by ^36^ and ^35^ differences in HbA1c between trimester groups similar to those observed in our study have been reported using both parametric ^35^^,^^36^ and non-parametric analyses, ^33^^,^^34^^,^^38^ suggesting that these findings are consistent across analytical methods.

Our study provides population-specific HbA1c reference intervals for Ethiopian non-diabetic pregnant women, which may assist in the clinical interpretation of HbA1c across gestation. These values could be particularly useful in settings where access to oral glucose test was limited.

### Limitations

These findings need to be interpreted with regard to a number of issues. Participants were recruited consecutively from urban antenatal clinics limiting generalizability to rural populations. In addition, iron status was not assessed using biomarkers such as serum ferritin or transferrin saturation. Future studies incorporating these measures would allow confirmation of normal iron stores and facilitate the development of reference intervals in truly iron-replete pregnant women. Although previous evidence suggests that the influence of mild anemia and iron supplementation on HbA1c is clinically small, ^49^ the potential impact of more severe iron deficiency remains uncertain and warrants further investigation.

Inflammatory markers (e.g., CRP) were not measured, and chronic low-graded or subclinical inflammation may influence HbA1c; thus, residual confounding cannot be excluded. Hemoglobin variants (e.g., Sickle cell trait and thalassemia) were not directly screened due to resource constraints; however, no history of hemoglobin disorders was identified during screening, and the Cobas c311 TINIA assay is analytically resistant to common structural variants, making significant confounding unlikely.

In terms of sample size, the sample size in the third-trimester group after the data processing was 121 (with the minimum recommendation set by IFCC/CLSI for estimation of reference intervals). While this subgroup was smaller than the first and second-trimester groups, and close to the suggested minimal for good precision, the precision analysis showed narrow 95% Confidence Intervals (around 0.16% HbA1c) for both the lower reference limit and upper reference limit, indicating good statistical precision. Importantly, the 95% Confidence Interval for the upper reference limit (5.82%–5.98%) was close to the existing clinical thresholds, so the observed statistical uncertainty is probably not translating into clinically relevant ambiguity in the interpretation. In addition, the good level of agreement between the parametric and non-parametric reference intervals, as only 0.1% differences was found at the third-trimester, showed that the results of the smaller sample size did not affect the robustness of the estimated reference interval for HbA1c at the third trimester. Nevertheless, studies including larger third-trimester cohorts would further improve the precision of the estimated reference limits and strengthen their generalizability.

Additionally, due to the cross-sectional design, causal inference and within-individual changes overtime cannot be established.

### Clinical implications and generalizability

The findings of this study provide important context-specific reference intervals for HbA1c by trimester in pregnancy in an Ethiopian population. These results demonstrate a clear trimester-dependent pattern, highlighting the importance of gestational age when interpreting HbA1c values.

In Ethiopia, HbA1c interpretation during pregnancy has often relies on non-pregnant reference intervals or lacks trimester-specific reference interval, which may not accurately reflect physiological variation during different stages of gestation. The present findings contribute to addressing this gap by providing population-specific data.

In many low-resource settings, including Ethiopia, access to oral glucose tolerance testing remains limited due to cost, patient burden, and laboratory constraints. In this context, glycated hemoglobin testing, if available, may be a practical alternative to measure glycemic status during pregnancy. Reference intervals that are derived locally and are trimester-specific may thus aid in more accurate interpretation and in better clinical decision-making.

This reference interval may prove to be useful in pregnancy to aid interpretation of HbA1c, but its clinical utility remains to be assessed. For this, prospective studies should be conducted to determine the effect of their use on the detection of GDM, glycemic monitoring, and pregnancy outcomes.

In addition, considerations of feasibility and cost-effectiveness are essential before integration into routine clinical practice in resource-limited settings.

The reference intervals are most directly applicable to non-diabetic pregnant women of Ethiopian descent receiving antenatal care in urban setting. The observed differences in HbA1c values between trimesters are consistent with global evidence, supporting the biological plausibility of the findings. However, differences in absolute HbA1c values across populations suggest that these intervals should not be generalized to other ethnic or geographic groups without further validation.

## Conclusion

This study establishes trimester-specific HbA1c reference intervals for non-diabetic pregnant women in Ethiopia, contributing population specific data to inform clinical interpretation during pregnancy. The established intervals (4.8–5.9% in the first, 4.2–5.8% in the second, and 4.5–5.9% in the third trimester) demonstrate significance differences in HbA1c between trimester groups, with lower values in the second trimester and relatively higher value in the first and third trimester.

These finding do not support the use of a single static reference interval during pregnancy and emphasize the importance of trimester-specific interpretation.

## Recommendations

1. Policy and laboratory practice: The Ethiopian Federal Ministry of Health and laboratory directors should recognize the clinical utility of HbA1c and integrate it into routine antenatal care pathways, where feasible.
2. Clinical Practice: Clinicians should interpret HbA1c values in non-diabetic pregnant women using locally derived, trimester-specific reference intervals to improve diagnostic accuracy and support appropriate clinical-decision making.
3. National Capacity: We recommend that the Ethiopian Public Health Institute and the Federal Ministry of Health prioritize a national multicenter study to establish locally validated reference intervals for HbA1c and other key laboratory parameters, with particular emphasis on pregnant populations across diverse geographic regions.

## Author declaration

The authors declare: That the article is the author’s original work. The article has not received prior publication and is not under consideration for publication elsewhere. That all authors have seen and approved the manuscript being submitted. The authors abide by the copyright terms and conditions of Elsevier and Clinics.

## CRediT authorship contribution statement

N.C.: Writing-review & editing, Writing-original draft, Validation, Software, Methodology, Investigation, Formal analysis, Conceptualization. R. L., G. G., C. Z. and C. W.: data acquisition, software, and formal analysis. D. C.: methodology, funding acquisition, supervision, and writing-review and editing.

## Ethical approval

This study was conducted in accordance with the principles of the Declaration of Helsinki. Ethical approvals obtained from the Institutional Research Ethics Committee (IRERC) of Addis Ababa University (Protocol no.014/24/Physio). Written informed consent was obtained from all participants prior to enrollment in the study.

## AI use declaration

AI was not used in the preparation of this work

## Consent for publication

Not applicable.

## Relater work

We wish to disclose a related submission entitled “Reference Interval of HbA1c for a non-diabetic non-pregnant women, Addis Ababa, Ethiopia: A Cross-sectional study” submitted to the Pan Africa Medicine Journal. That study focuses on non-pregnant women, while the present work extends the investigation to established trimester-specific reference intervals in pregnancy.

## Abbreviations

GDM, Gestational Diabetes Mellitus.

HbA1c, Glycated hemoglobin.

## Funding

This research received no specific grant from funding agencies in the public, commercial, or not-for-profit sectors.

## Declaration of competing interest

The author declares that they have no financial or personal relationships that could have influenced the research reported in this paper.

## Data Availability

The datasets generated and/or analyzed during the current study are available from the corresponding author upon reasonable request.
