# Supplementary material for: Trimester-specific reference interval for glycated hemoglobin in non-diabetic pregnant women: A cross-sectional study
Source: Clinics (Sao Paulo). 2026 Sep 19;81:101160. doi: 10.1016/j.clinsp.2026.101160 (PMC13628227; doi:10.1016/j.clinsp.2026.101160)
Supplement: Supplementary file 1 [file mmc1.pdf]

Title: Trimester-Specific Reference Interval for Glycated Hemoglobin in Non-Diabetic Pregnant Women: A Cross-sectional Study in Addis Ababa, Ethiopia.

Completed STROBE Checklist for Our Cross-Sectional Study

Item NO	Recommendation	Page No/Section/Note
**Title and Abstract**<br>1.	(a) Indicate the study design with a commonly used term in the title or the abstract<br>(b) Provide in abstract an informative and balanced summary of what was done was and what was found	**Abstract** ("Methods A cross-sectional study…."<br><br>**Abstract (**The entire abstract provides a balanced summary of backgrounds, key results, and conclusion.)
**Introduction**<br>**2.**	Explain the scientific background and rational for the investigation being reported.	**Introduction (**Paragraph 1-5, and 7)
3.	State specific objective, including any prespecified hypothesis	Introduction (Final paragraph before "Materials and Method": "Therefore, this study aims to…We hypothesized that…"
**Methods**<br>**4.**	Present key elements of study design earlier in the paper	**Methods (**Study design: "A Cross-Sectional Observational Study was conducted…..")
**5.**	Describe the setting, locations, and relevant dates, including period of recruitment, exposure, follow-up, and data collection.	**Methods (**Study setting: "Janmeda and Teklehaymanot Health Centers…", "From February 4 to July 18, 2025.")
**6.**	Give the eligibility criteria, and the source and methods of selection of participants	Methods (Participants: "Source population" "Eligibility criteria [inclusion/exclusion lists], "Consecutively recruited").
**7.**	Clearly define all outcomes, exposures, predictors, potential confounders, and effect modifiers. Give diagnostic criteria, if applicable.	**Methods (**Variables and measurements: Define HbA1c (outcomes), trimester (exposure), other predictors, confounders, and GDM diagnostic criteria (IADPSG)
**8.**	For each variable of interest, give sources of data details of methods of assessment (measurement). Describe	**Methods (**Data sources and management: Detailed for each variable. "Comparability of assessment

	comparability of assessment methods if there is more than one group	method" Paragraph explicitly addresses this.)
**9.**	Describe any efforts to address potential sources of bias	**Methods (**Assessment of Bias: Dedicated section on section, measurement, and confounding bias.)
**10.**	Explain how the study size was arrived at	**Methods** (Sample size: Explicitly states calculations based on IFCC guidelines, target of 120 per group.)
**11.**	Explain how quantitative variables were handled in the analyses. If applicable, describe which groupings were chosen and why	**Methods** (Statistical Analyses: Describes normality tests, parametric summary, trimester groupings, and outlier detection.)
**12.**	(a)describe all statistical methods, including those used to control for confounding	**Methods** (Statistical Analysis: Describes ANOVA, Post-hoc tests. Confounding addressed by exclusion criteria, not statistical adjustment)
	(b)Describe any methods used to examine subgroup and interaction	**Methods** (The entire analysis is subgroup (trimester) based. ANOVA and Post-hoc tests are described.)
	(c)Explain how missing dada were addressed	**Methods** (Statistical Analysis: participants with missing data for the primary outcomes….were excluded from the analysis…..)
	(d)Cross-sectional study –if applicable, describe analytical methods taking account of sampling strategy	**N/A** (Consecutive sampling was used; no complex sampling strategy)
	(e)Describe any sensitivity analysis	**N/A** (Not mentioned in the report)
**Results**		
**13.**	(a)Report number of individuals at each stage of study	**Results** (Participants: "A total of 524…assessed…100 were excluded…final…424..) and figure 1
	(b)Give reason for non-participation at each stage	**Results** (Participants: List specific reasons for exclusion.)
	(c)Consider use of a flow diagram	**Results** (Figure 1 is the participants' flow diagram.)
**14.**	(a)Give characteristics of study participants and information on exposure and potential confounders	**Results** (Table 1 and Table 2 provide detailed participant characteristic by trimester.)
	(b)Indicate number of participants with missing data for each variable of interest	**Results** (Maternal clinical characteristic: "There were no missing data for the variables reported".)

**15.**<br><br>**16.**<br><br><br><br><br><br>**17.**	Cross-Sectional Study–Report number of outcome events or summary measures<br>(a)Give unadjusted estimate and, if applicable, confounder-adjusted estimate…<br><br>(b)Report category boundaries when continuous variables were categorized<br>Report other analysis done–e.g. Analysis of subgroups, and sensitivity analysis	**Results** (Table 3: Reports n, mean, SD, and reference interval for HbA1c for each trimester.)<br>**Results** (Table 4 & 5: Report unadjusted mean differences and 95% CI from ANOVA and Post-hoc tests.)<br>**Results** (Variables: Defines: trimester category boundaries.)<br>**Results** (The comparison of HbA1c across trimester (ANOVA, post-hoc) is the primary "Other analysis" reported.)
**Discussion**<br>**18.**<br><br>**19.**	Summarize key results with reference to study objective<br><br><br>Discuss limitation of the study, taking into account sources of potential bias or impression. Discuss both direction and magnitude of any potential bias.	**Discussion** (Key results: First paragraph summarizes key RIs and pattern, referencing the study's aim.<br>**Discussion (**Limitations: Discusses generalizability)
**Recommendation**<br>**20.**<br><br>**21.**	Give a cautious overall interpretation of results considering objectives, limitations, multiplicity of analysis, results from similar studies, and other relevant evidence<br>Discuss the generalizability (external validity) of the study result	**Discussion (**Interpretation: Interprets findings in context of physiology and global literature, acknowledges ethnic variation.)<br>**Discussion** (Clinical Implications and generalizability; explicitly states the population to which the results are generalizable.)
**Other information**	Give the source of funding and the role of the funders for the present study on which the present article is based	This research did not receive any specific grant from funding agencies in the public, commercial or non-for-profit sectors.
